# Evolution of a Sigma Factor: An All-In-One of Gene Duplication, Horizontal Gene Transfer, Purifying Selection, and Promoter Differentiation

**DOI:** 10.3389/fmicb.2016.00581

**Published:** 2016-04-25

**Authors:** Gamaliel López-Leal, Miguel A. Cevallos, Santiago Castillo-Ramírez

**Affiliations:** ^1^Programa de Inmunología Molecular Microbiana, Departamento de Microbiología y Parasitología, Facultad de Medicina, Universidad Nacional Autónoma de MéxicoMexico City, Mexico; ^2^Programa de Genómica Evolutiva, Centro de Ciencias Génomicas, Universidad Nacional Autónoma de MéxicoCuernavaca, Mexico

**Keywords:** molecular evolution, sigma factors, gene duplication, horizontal gene transfer, promoter differentiation, purifying selection

## Abstract

Sigma factors are an essential part of bacterial gene regulation and have been extensively studied as far as their molecular mechanisms and protein structure are concerned. However, their molecular evolution, especially for the alternative sigma factors, is poorly understood. Here, we analyze the evolutionary forces that have shaped the *rpoH* sigma factors within the alphaproteobacteria. We found that an ancient duplication gave rise to two major groups of *rpoH* sigma factors and that after this event horizontal gene transfer (HGT) occurred in *rpoH*_1_ group. We also noted that purifying selection has differentially affected distinct parts of the gene; singularly, the gene segment that encodes the region 4.2, which interacts with the −35 motif of the RpoH-dependent genes, has been under relaxed purifying selection. Furthermore, these two major groups are clearly differentiated from one another regarding their promoter selectivity, as *rpoH*_1_ is under the transcriptional control of σ^70^ and σ^32^, whereas *rpoH*_2_ is under the transcriptional control of σ^24^. Our results suggest a scenario in which HGT, gene loss, variable purifying selection and clear promoter specialization occurred after the ancestral duplication event. More generally, our study offers insights into the molecular evolution of alternative sigma factors and highlights the importance of analyzing not only the coding regions but also the promoter regions.

## Introduction

Bacteria face many different environmental challenges and a key element to cope with this is the capacity to modulate gene expression. There are several ways in which a bacterium can regulate gene expression, one of them being the use of different sigma factors to rapidly mount a response to environmental changes. Bacteria have one essential housekeeping sigma factor, which controls the transcription of many genes, and some species additionally might have one or more alternative sigma factors that promote very specific sets of genes required for particular stress conditions (Helmann, 2002; Gruber and Gross, 2003; Osterberg et al., 2011; Feklistov et al., 2014). One of these alternative sigma factors is σ^32^, which is encoded by the *rpoH* gene; this sigma factor plays a key role in the heat shock stress response in many bacteria (Gruber and Gross, 2003; Osterberg et al., 2011; Feklistov et al., 2014). Many of the heat-shock proteins that define the heat-shock stimulon are regulated by σ^32^. This sigma factor is found as a single copy gene in *Escherichia coli*, as well as in other gammaproteobacteria (Bukau, 1993); nonetheless, it is has been found as a multiple copy gene in many alphaproteobacteria genomes (Narberhaus et al., 1997; Galibert et al., 2001; Kaneko et al., 2002; Gonzalez et al., 2006; Green and Donohue, 2006; Martinez-Salazar et al., 2009a). For instance, two *rpoH*-like genes have been found in *Rhodobacter sphaeroides* (Green and Donohue, 2006), whereas *Bradyrhizobium japonicum* has three copies (Narberhaus et al., 1997). In the case of *Rhizobium etli*, which has two copies, one of them, namely *rpoH*_1_, deals with the heat-shock response, whereas the second copy (*rpoH*_2_) is needed for the osmotic-shock response (Martinez-Salazar et al., 2009a). This trend seems to apply to other alphaproteobacteria, where one of the *rpoH* genes (generally named *rpoH*_1_) is involved in the heat-shock response, whereas the other (s) copies are involved in other stress conditions (Narberhaus et al., 1997; Green and Donohue, 2006). Although in alphaproteobacteria the different *rpoH* genes are differentially regulated, it has also been found that they can partially or completely complement the temperature-sensitive phenotype of an *E. coli rpoH* mutant (Narberhaus et al., 1997; Green and Donohue, 2006; Martinez-Salazar et al., 2009a). This might suggest that the functional properties of the different RpoH proteins coded in these genomes are not that different after all. Despite this, it has also been shown that different RpoH copies target a common shared set of genes within a genome but also each copy has a set of exclusive targets (Dufour et al., 2012). This implies that although the functions of the RpoH copies may be similar, they are not exactly the same. It is possible that, to some extent, the differentiation between these copies could lay within the parts of the protein that recognize the upstream regions of the genes regulated by these RpoH sigma factors.

Sequence analyses have shown that RpoH sigma factors form a monophyletic group and belong to one of the main four groups in which the σ^70^ family is divided into (Helmann, 2002; Paget and Helmann, 2003). The RpoH sigma factors are within the 3rd group of this family and are distantly related to the first group, which is composed of essential housekeeping factors σ^70^. This third group has been implicated in stress responses and developmental programs such as flagella biosynthesis or sporulation (Helmann, 2002; Paget and Helmann, 2003). The RpoH sigma factors have two conserved amino acid regions, namely 2.4 and 4.2, that contact the promoter sequences (2.4 contacts the −10 motif, whereas 4.2 interacts with the −35 motif) of the genes controlled by these sigma factors; the 4.2 resides in the carboxy-terminal section of the protein, whereas region 2.4 lies within the amino-terminal part of the protein (Feklistov et al., 2014). Compared to other members of the σ^70^ family, the RpoH sigma factors are characterized by a region of highly conserved amino acids—involved in RNA polymerase interactions—that is known as the “RpoH box” (Nakahigashi et al., 1995). Previous sequence analyses have focused on the whole σ^70^ family mainly at the level of protein sequences. To date, no single study has focused on the homologous genes from the *rpoH* sigma factor subfamily. Most studies instead have either grouped the sigma factors into different subgroups and/or determined which parts of the protein sequences were more conserved. Notably, much less is known about the evolutionary forces that have shaped the genetic variation of the genes that code for these proteins. For instance, there has been little attention paid to the role of natural selection (either purifying or diversifying) as a potential major evolutionary driver for the sigma factors or the impact of horizontal gene transfer (HGT) on these genes.

Nowadays, with the availability of so many bacterial genomes, evolutionary studies can be carried out much more easily than a couple of decades ago, when the shortage of sequences was one of the major shortcomings. Therefore, given the scarcity of evolutionary studies concerning sigma factors, the aim of our work was to study the molecular evolution of the *rpoH* sigma factors. Our results show that this family has a very particular evolutionary history, where after an ancient gene duplication event—with subsequent gene loss and HGT events—variable purifying selection across the gene and functional differentiation of the promoter regions have occurred.

## Materials and methods

### Genomes used and RpoH homologous genes

To carry out our study 53 bacterial genomes were considered (Additional Material 1), 46 of them belong to the alphaproteobacteria; however, we also included 7 gammaproteobacteria genomes. We chose these 46 alphaproteobacteria genomes as previous phylogenomic analyses have established the species tree for them (Gupta and Mok, 2007; Castillo-Ramirez and Gonzalez, 2008) and this was relevant for some of the phylogenetic analyses that we conducted. Furthermore, these genomes cover the 7 main orders, and many families, of the alphaproteobacteria. These genomes were downloaded between November and December 2015 from the NCBI and are listed in Additional Material 1. We used the RpoH protein from *E. coli* K12 as a seed in the blast searches (Altschul et al., 1990), setting an *E*-value cutoff of 1.0e-15, against the proteomes encoded by the other genomes. All the proteins that showed an *E*-value lower than the cutoff and that aligned at least 70% of their length were kept for further analysis. Then, a protein Multiple Sequence Alignment (MSA) was created through MUSCLE (Edgar, 2004), specifying 20 iterations. To create a DNA alignment in frame, we used the program TRANALING, which is part of the EMBOSS suite (Rice et al., 2000), that aligns coding regions based on the based on the corresponding translations of the genes considered.

### Phylogenetic inferences and among-*rpoH* divergence

We conducted a Maximum Likelihood (ML) phylogeny on the protein MSA and chose the most adequate model using ProtTest (Abascal et al., 2005) that in this case was: LG (as the model of amino acid substitution), a correction for among site variation (G) and allowed a proportion of invariable sites (I). We set a non-parametric bootstrap analysis (100 replicates) to establish the support for the clades. In order to have a proxy for the species tree of the species of the genomes here analyzed, we considered the set of 31 orthologs used by Ciccarelli et al. (2006) to reconstruct a tree of life. Of the 31 orthologs we only kept those cases that are single gene families (only one gene per genome) and that are also present in each of the genomes employed in this study; this reduced the number of cases to 8 orthologs (see Additional Material 2). None of these genes had signals of recombination. The protein alignments of these genes were concatenated and, on this concatenate alignment, a Maximum Likelihood phylogeny was constructed. We ran the ML phylogeny with the model LG+G+I, as determined by ProtTest (Abascal et al., 2005), this phylogeny is provided in Additional Material 3.

We ran several topology tests to establish whether or not the topology obtained for the proxy for the species tree provides an equivalent explanation for the two main groups identified in the RpoH family. For this analysis, we considered the RpoH sequences from 15 genomes (Additional Material 4) that have one copy in each one of both RpoH groups. The protein phylogenies for these two groups were conducted via PhyML (Guindon et al., 2010), we carried out statistical model selection as before, via ProtTest (Abascal et al., 2005). The best models in these cases were as follows: JTT+G+I+F for RpoH_1_ and LG+G+I+F for RpoH_2_. We conducted two topology tests, the Kishino-Hasegawa (KH) and the Shimodaira-Hasewaga (SH), by means of the program codeml (seqtype = 2), from PAML (Yang, 2007). We ran codeml considering the appropriate substitution rate matrix and among site rate variation estimating the alpha parameter for the gamma distribution. Two topologies were tested in each case, one was the ML topology obtained for the proxy for the species tree and the other was the ML phylogeny of either RpoH_1_ or RpoH_2_. The trees used for this analysis are shown in Additional Material 9.

We also ran a molecular dating analysis on the RpoH protein MSA, using the Bayesian Evolutionary Analysis by Sampling Trees (BEAST) program (Drummond and Rambaut, 2007). We employed an uncorrelated log-normal relaxed clock with site model specifications equal to those of the RpoH ML phylogeny. We calibrated the clock using information from the TimeTree database (Hedges et al., 2006, 2015); we used the dates of the most recent common ancestor of (1) the Enterobacteriales (425 million years ago [mya]), (2) the gammaproteobacteria (1787.2 mya), and (3) the gamma and alphaproteobacteria (2472.1 mya). We specified normal prior distributions on the calibrated nodes centered at the values mention above and with 20 standard deviations. The analysis was run twice for 50,000,000 generations, sampling every 5000 generations and discarding the first 12,500,00 generations as burn-in. We are confident about our results as the Effective Sample Size for all the parameters—except for the calibrated Yule model that was 195.176—were >200 and because the 2 BEAST runs converged on similar posterior distributions.

### Selection analysis and Tajima'S relative test

All the selection analyses were run using codeml from PAML (Yang, 2007). We tested for variability of selection (type and magnitude) across the codons of the gen using some site models; three pairs of models were employed. The first pair considers M0 (just one dN/dS ratio) and M3 (“n” discrete categories of dN/dS) and has 4 degrees of freedom (df); the second pair includes M1a (two classes of codons, conserved [dN/dS < 1] and neutral [dN/dS = 1]) and M2a (the same as M1a plus another category that allows for dN/dS > 1), this has 2 df; and the third pair encompasses M7 (a beta distribution that allows dN/dS to vary in the interval [0, 1]) and M8 (the same beta distribution as in M7 but adding an extra class for codons with dN/dS >1), with 2 df. We used MEGA6 (Tamura et al., 2013) to conduct the Tajima's relative test (Tajima, 1993) to evaluate the hypothesis of equal rates between two *rpoH* groups. The analysis involved the amino acid sequences of the two copies of *R. etli* CFN42 and *E. coli* K-12 as an out group. Similar analyses were carried out with the two RpoH copies of *B. abortus* 9-941, *Jannaschia* sp. CCS1, and *R. sphaeroides* 2.4.1 in all these cases also using *E. coli* K-12 as an out group.

### Promoter analysis

To carry out promoter identification, we used the upstream regions of *rpoH*_1_ and *rpoH*_2_ from a set of *Rhizobiales* genomes (Additional Material 6). For both *rpoH*_1_ and *rpoH*_2_, two data sets were created collecting the upstream non-coding regions using a custom PERL script. One set contained the first upstream 150 nucleotides from the ATG that defines the translation start site, whereas the other contained the whole upstream region until the next gene. We used RSAT, Regulatory Sequence Analysis Tools (Medina-Rivera et al., 2015), to conduct promoter identification on these data sets. First, by means of spaced dyads analysis *de novo* promoter identification was conducted. Different motifs were created with a dyad spacing parameter of 13–22, other than this default parameters were used (Additional Material 7). However, in the case of the *rpoH*_2_ group we used a minimum weight of 6 for the assembled motifs. Alternatively, we also used matrices for promoter identification. We created matrices employing the program info-gibbs also from RSAT and using the information of previously reported promoters for alphaproteobacteria (Martinez-Salazar et al., 2009a,b; Barnett et al., 2012; Jans et al., 2013; Schluter et al., 2013). The matrices were constructed independently for the −35 and −10 promoter motifs—when considering the σ^70^ family promoters class (SigA, RpoH_1_, RpoH_2_, RpoE_2_, RpoE_4,_ and RpoE_1_)—and −24 and −12 motifs for the σ^54^ family promoters class. We used the matrices to locate potential promoter motifs using matrix-scan (also from RSAT) with a *p*-value cutoff of 0.001 for the −35 motifs and in some cases a *p*-value of 0.0025 for the −10 motifs, we used different *p*-values as it has been shown that the −10 motif tends to be less conserved (Ramirez-Romero et al., 2006). In the case of the potential promoter that belonged to σ^70^ family, we retained all the cases that had a spacer of 13–23 nt between −35 and −10 motifs (Additional Material 8).

## Results

### A mixture of gene duplication, HGT, and gene loss events

**Figure 1:**
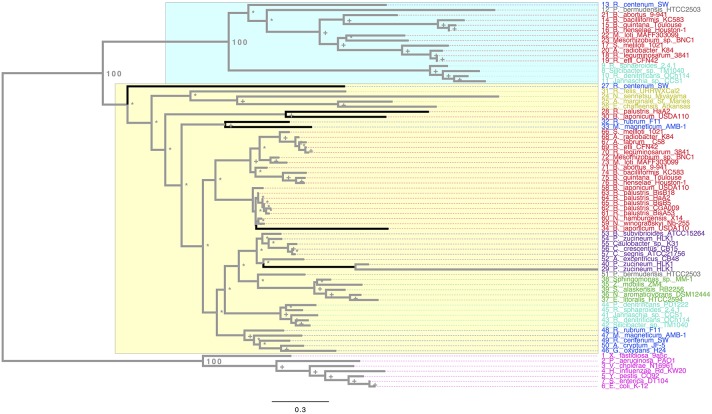
**Maximum Likelihood phylogeny of the RpoH**. The ML phylogeny is based on the protein alignment. We colored the RpoH sequences to denote the different orders of the alphaproteobacteria: red, *Rhizobiales*; light blue (cyan), *Rhodobacterales*; green, *Sphingomonadales*; blue, *Rhodospirillales*; yellow, *Rickettsiales*, gray, *Parvularculales*; violet, *Caulobacterales*, and magenta for the gammaproteobacteria. The numbers next to some nodes are the bootstrap values for some of the main groups, plus signs show nodes with 70 or higher bootstrap support, whereas asterisks denote nodes with less than 70 bootstrap support. The black branches denote HGT events. The yellow rectangle shows all the sequences within the *rpoH*_1_ group, whereas the blue one covers the sequences composing the *rpoH*_2_ group. The scale bar describes the number of substitution per site.

In order to understand the molecular evolution of the *rpoH* genes we used more than 50 genomes (see Additional Material 1), most of which are classified as alphaproteobacteria, although we also considered few gammaproteobacteria genomes as outgroup. Notably these genomes represent the main orders of the alphaproteobacteria (videlicet *Rhizobiales, Rhodobacterales, Sphingomonadales, Rhodospiralelles, Rickettsiales, Parvularculales, Caulobacterales*). We first wanted to quantify the number of RpoH homologs per genome and, therefore, we conducted BLAST searches using the RpoH sequence from *E. coli* K12 as seed to identify the RpoH protein sequences in the rest of the genomes (see Section Materials and Methods). A total of 76 RpoH protein sequences were found and to visualize their phylogenetic relationships we constructed a Maximum Likelihood (ML) phylogeny (Figure 1). We first note that, whereas there is only one copy of RpoH in the gammaproteobacteria genomes (pink labels, Figure 1), there are many alphaproteobateria that have more than one copy per genome (light blue, blue, gray, and red labels, Figure 1). For example, *R. etli* CFN42 has two copies and the same applies for other Rhizobia such as *S. meliloti* or *M. loti* but also for species from the genera *Bartonella* and *Rhodobacter*. The most extreme case is *B. japonicum* USDA, which shows three copies; at the other extreme—with just one copy—there are some strains (CGA009, BisA53, BisB5, and BiisB18) from the species *Rhodopseudomonas palustris* but also *S. alaskensis* RB2256, *Z. mobilis* ZM4, *E. litoralis* HTCC2594, and *Sphingomonas_*sp. MM-1 (green labels). Notably, all the alphaprotebacteria RpoH sequences form a monophyletic group, which is very well supported (i.e., a bootstrap value of 100, see Figure 1) and well differentiated from the gammaproteobacteria RpoH sequences. Furthermore, the RpoH sequences from the alphaproteobacteria and gammaproteobacteria do not mix together whatsoever. This implies that there has been no HGT between these two classes (alphaproteobacteria and gammaproteobacteria). Together these data suggest that the presence of more than one *rpoH* gene per genome is a common occurrence in the alphaproteobacteria and that HGT involving these genes has no occurred between the alphaproteobacteria and gammaproteobacteria.

In order to a have a reference to identify HGT and duplication events, we constructed a proxy for the species tree of the genomes considered here using a previous set of orthologous genes (see Section Materials and Methods and Additional Material 2). Given this proxy for the species tree (see Supplementary Material 3), the positions of some sequences on the RpoH tree are better explained as cases of HGT; these are marked on the tree (see black branches). Such is the case of one of the RpoH copies of *Rhodospirillum centenum SW*, which clusters basally to the Rickettsiales. Another two cases are two of the copies of *B. japonicum* USDA and one of the copies of *Rhodopseudomonas palustris* HaA2. However all these cases of HGT seem to have occurred within the alphaproteobacteria. On the other hand, Figure 1 clearly shows that there are two main groups (*rpoH*_1_, yellow rectangle, and *rpoH*_2,_blue rectangle) in the alphaproteobacteria group; notably, the phylogenetic relationships among the sequences within the two groups—if one does not take into account the HGT events previously mentioned—are similar to those found in the species trees of the Rhizobiales order (Castillo-Ramirez and Gonzalez, 2008) and to our proxy for the species tree. This might suggest that a duplication of the *rpoH* gene might have occurred some time ago and then each copy has accumulated changes that reflect a history of the species. To gain further insight into this, we tested whether the topologies for a common set of taxa (see Additional Material 4) within the two groups where similar to that of the proxy for the species tree (see Section Materials and Methods). We did not find significant differences (at an alpha of 0.01) between the topology of either group and that of the proxy for the species tree (see Table 1); this was true irrespective of the test used—we employed the Kishino–Hasegawa test but also the Shimodaira–Hasewaga test. From these results we conclude that the phylogenetic relationships within each group (not taking into account the HGT events in *rpoH*_1_ group) are pretty similar to those of the species tree, which further corroborates the view that a duplication event gave rise to the two groups. Of note the HGT events seemed to be found exclusively in the *rpoH*_1_ group. Although, a duplication event has generated two *rpoH* groups, the genes belonging to each one of them have no been equally conserved; clearly, the *rpoH*_1_ group had more taxa than the *rpoH*_2_ group (see Figure 1). Actually, whereas all the alphaproteobacteria had one member of the *rpoH*_1_ group, not all the alphaproteobacteria had a member of the *rpoH*_2_ group. For instance, the two species from the genus *Nitrobacter*, many *R. palustris* strains, *A. tumefaciens* C58, *S. alaskensis* RB2256, *Z. mobilis* ZM4, *E. litoralis* HTCC2594, and *Sphingomonas_*sp. MM-1 just had one *rpoH* homolog and in all the cases it belongs to the *rpoH*_1_ group. This implies that several gene loss events have occurred over time but these have only affected the members of the *rpoH*_2_ group.

**Table 1:** **Topology tests**.

**Gene**	**pKH^+^**	**pSH^+^**
RpoH1	0.500	0.756
RpoH2	0.043	0.061

*Topology tests to establish whether the topology obtained for species tree provides an equivalent explanation for RpoH1 and RpoH2 alignments*.

+ *p-values under the Kishino–Hasegawa (KH) test and the Shimodaira–Hasewaga (SH) test, respectively*.

**Figure 2:**
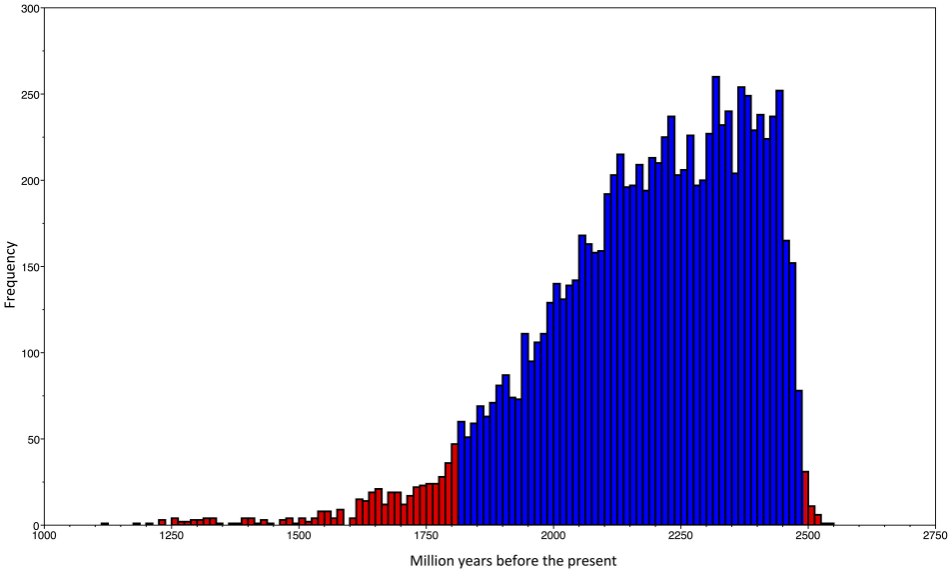
**Dating of the duplication event**. Frequency histogram of the traces of the origin of the duplication event that gave rise to the two *rpoH* groups.The blue bars show the 95% highest posterior density (HPD) interval, whereas the red bars give all the values not included within the HPD.

Our ML phylogeny suggests that the duplication event that gave origin to the *rpoH*_2_ group is rather ancient; therefore, in order to further explore this, we constructed a calibrated phylogeny using BEAST (Drummond and Rambaut, 2007; see Section Materials and Methods; Additional Material 5). Our calibrated phylogeny suggests that the duplication occurred some 2214 million years ago (mya) (Figure 2), 95% highest posterior density interval (HPD) 1812–2491 mya—the whole calibrated phylogeny is provided in Additional Material 5. Therefore, our dating analysis tells us that this duplication event is ancient; more than 1800 mya even if one takes the lower bound (1812 mya) of the 95% HPD. To summarize, the ML phylogeny, the topology tests and the molecular dating analysis indicate that an ancestral duplication of the *rpoH* gene occurred some 2214 mya within the alphaproteobacteria, after the split between the gamma and the alphaproteobacteria, and yielded two *rpoH* groups. Furthermore, whereas the *rpoH*_1_ group has experienced cases of HGT no such cases were found in the *rpoH*_2_ group. However this latter group appears to have endured several instances of gene loss.

### Variable purifying selection along the *rpoH* gene and similar amino acid rates between the *rpoH* groups

**Figure 3:**
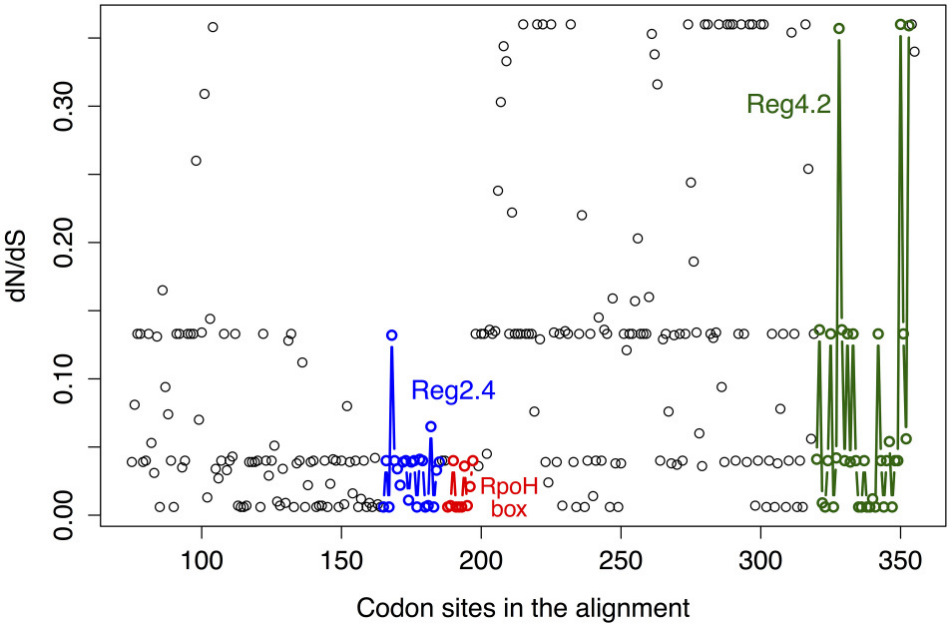
**Variability in dN/dS among codons**. Mean posterior probability values of dN/dS for the codon alignment. These values depend on the maximum likelihood estimates for dN/dS under the M3 model and were calculated via the Naïve Empirical Bayes method. The color code shows regions codifying some relevant parts of the RpoH protein and is as follows: blue circles, region 2.4 that interacts with the −10 motif of the RpoH-dependent genes; red circles, conserved region that is particular to *rpoH* genes; green circles, region 4.2 that interacts with the −35 motif of the RpoH-dependent genes.

Next, we wanted to know if any of the *rpoH* groups have signals for positive selection and to that end we used the ratio dN/dS (see methods). This ratio is commonly used to infer the type and intensity of selection: where dN/dS >1 implies diversifying (positive) selection; dN/dS < 1 indicates purifying (negative) selection and dN/dS = 1 means neutral evolution. The program codeml implements different models of codon evolution and these models can be compared by means of likelihood ratio tests (LRT). Therefore, we employed this program to conduct several types of analysis to infer the type—and magnitude—of selection acting on these genes. We first obtained a Maximum Likelihood (ML) estimate of dN/dS for the whole gene. This value was 0.09446, which indicates a very strong purifying selection. However, this is an average estimate that cannot reflect how selection varies across time or among sites. Importantly, as different parts of a gene could be subject to different selection pressures, we also ran other models that allowed us to test for variability in dN/dS across codons; these are known as “site models” (Yang, 2007). We carried out three LRTs that allowed us to compare pairs of models as follows: (1) LRT for testing variable selection among codons (M0 vs. M3); (2) LRT for testing the presence of codons subject to positive selection (M1a vs. M2a); (3) alternative LRT to test for the presence of codons subject to positive selection (M7 vs. M8). The test for variable selection among codons (M0 vs. M3) was significant (LRT = 4347.887, prob = 0) implying that different parts of the gene have experienced different levels of purifying selection (see below). However, neither of the tests for codons subject to positive selection was significant: M1a vs. M2a LRT = 0 (prob = 1)—similar results were obtained with the other LRT (M7 vs. M8). Hence, we did not find any signals of codons under diversifying selection. To further explore how selection has changed across the gene we plotted the mean posterior probability values of dN/dS for every codon (see Figure 3). The level of purifying selection shows no constancy among the codons of the gene (see Figure 3), as there were some codons under very strong selection—values of dN/dS lower than 0.1 and approaching to 0-, whereas others were under weak purifying selection (dN/dS higher than 0.2). Furthermore, many sites (codons) in the amino-terminus of the protein seem to have experienced mild purifying selection as their dN/dS values are higher than 0.1 (see Figure 3). Notably, the codons that codify for the RpoH box and the region 2.4 were under strong purifying selection. On the other hand, we found clear evidence of relaxed purifying selection in the region 4.2—this is the region that interacts with the −35 motif of the RpoH-dependent genes. This analysis shows that strong purifying selection is the main type of selection. However, the intensity of purifying selection is not uniform along the gene.

In order to test the equality of amino acid evolutionary rate between the two *rpoH* groups, we conducted the Tajima's relative test (Tajima, 1993). For this test we employed the two copies of *R. etli* CFN42 and *E. coli* K-12 as an out group, the null hypothesis of equal rates between the *rpoH* groups was not rejected as the chi-square (chisq) test statistic was 0.49 (prob = 0.48384, 1 df). We also carried out Tajima's relative test using the two *rpoH* copies of other species—*B. abortus* 9-941 (chisq = 1.86, prob = 0.17245), *Jannaschia* sp. CCS1 (chisq = 3.46, prob = 0.06289), and *R. sphaeroides* 2.4.1 (chisq = 2.80, prob = 0.09426)—and again the null hypothesis of equality of evolutionary rate could not be rejected. Therefore, it seems that the amino acid evolutionary rate of RpoH has remained rather constant over time. To sum up, the dN/dS analysis indicates that although strong purifying selection is the main type of selection, its intensity is not uniform along the gene and the Tajima's relative test implies that similar amino acid evolutionary rate between the two *rpoH* groups.

### Different promoter organization for *rpoH*_1_ and *rpoH*_2_

**Figure 4:**
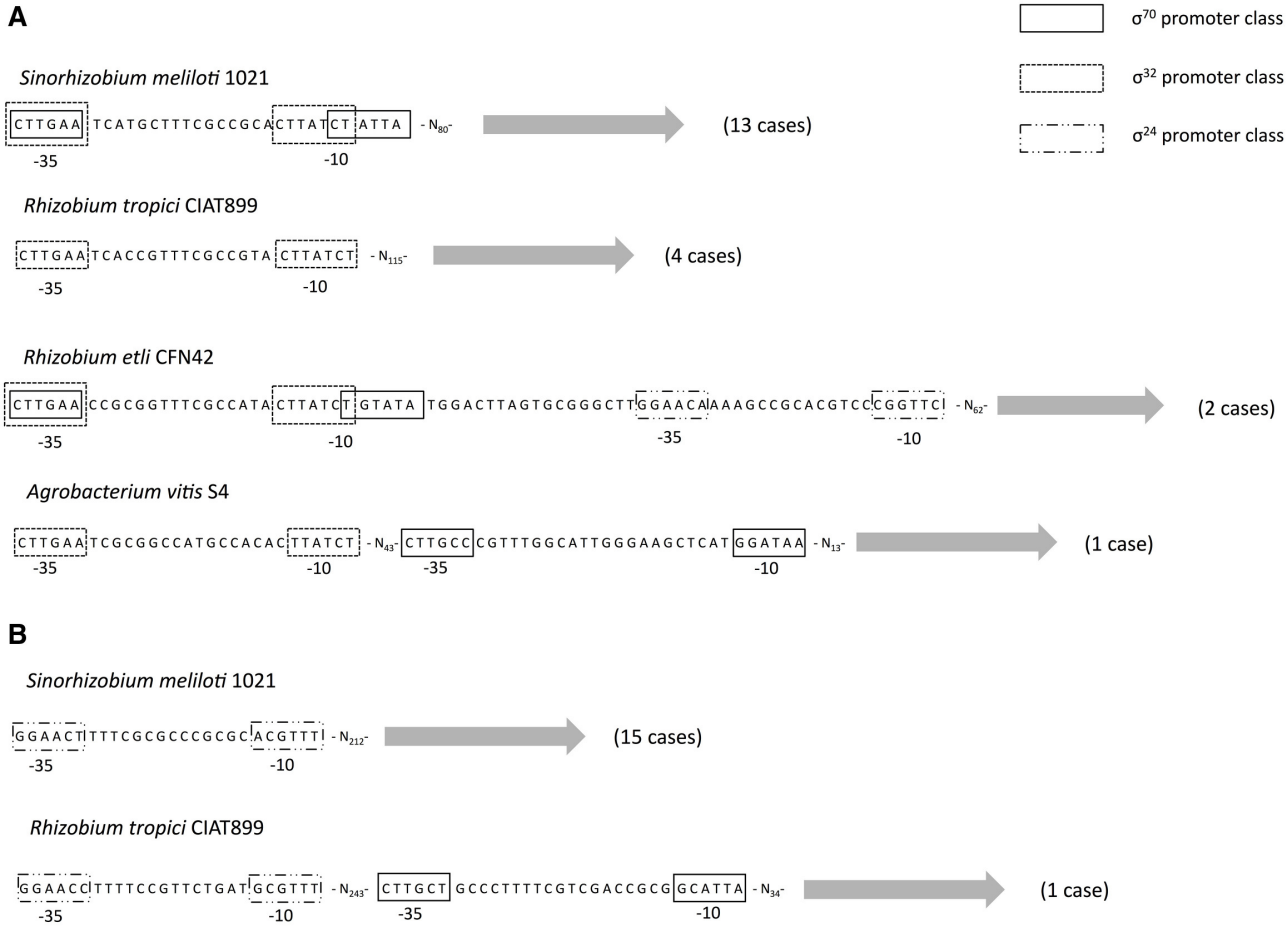
**Potential promoter organization**. Schematic representation of the potential promoter organization for *rpoH*_1_ **(A)** and *rpoH*_2_ **(B)**. Putative promoters classes for σ^70^, σ^32^, and σ^24^ are shown as boxes. The most common promoter organization is shown first—exemplified by *S. meliloti* 1021 in both cases **(A,B)**—and then the least frequent cases are shown below, in parentheses are given the number of strains that show such promoter organization.

Thus far, we have only considered the coding region of the gene—either the proteins sequences for the ML phylogeny, dating analysis, Tajima's relative test or DNA for the selection analysis—but a fundamental aspect of any gene is its transcriptional regulation. Therefore, to understand how the expression of *rpoH*_1_ and *rpoH*_2_ might have changed we conducted a promoter analysis. For this analysis we decided to focus only on the *Rhizobiales* order for two reasons: first, because non-coding regions are more difficult to align and, therefore, we restrained this analysis to a small evolutionary scale and, second, because previous experimental studies have mapped and characterize the promoters of these genes in some species from this order (Martinez-Salazar et al., 2009a; Barnett et al., 2012; Schluter et al., 2013). We used two approaches to identify the potential promoter signals (see Section Materials and Methods): first, we used a set of matrices constructed from promoters previously characterized (MacLellan et al., 2006; Martinez-Salazar et al., 2009b; Barnett et al., 2012; Schluter et al., 2013) and, secondly, we assembled *de novo* promoters from our set of upstream regions. The use of these two approaches allowed us to identify promoters that were highly represented (*de novo* promoters) and those that were poorly represented (using matrices). First, we found that all members from the *rpoH*_1_ group had promoter signals within the first 80–100 nt of the upstream region (see Figure 4), whereas most of the members of the *rpoH*_2_ group showed promoter signals far apart (>200 nt of the upstream region); the only exception was *R. tropici* CIAT899 which had promoter signals in the first 30–40 nt of the upstream region. Secondly, we noted that 75% of members of the *rpoH*_1_ group had promoter signals that indicate that it could be very likely under transcriptional control of σ^70^ and σ^32^ (see Figure 4). Notably, there is a considerably overlap between the promoter signals for σ^70^ and σ^32^. There were only four cases that just had promoter signals for σ^32^. In contrast, most members (80%) of the *rpoH*_2_ group only had promoter signals for just one sigma factor, namely σ^24^ (see Figure 4). A particular case is *R. tropici* CIAT899 that also had promoter signals for σ^70^ (see Figure 4). However, 4 strains did not show promoter signals for any of the sigmas that we considered; three of them are *R. etli* strains (MiM1, CFN42, and CIAT652) and *S. fredi* 257. Taken together these results indicate that very likely the two *rpoH* groups are under the control of different sigmas; even more, whereas the *rpoH*_2_ group has promoter signals for just one sigma, the *rpoH*_1_ group not only has promoter signals for two sigmas but these signals tend to overlap to a considerable extent. Additionally, the promoter signals are located at different distances for each *rpoH* group. In considering these results, it is clear that *rpoH*_1_ and *rpoH*_2_ likely have a different promoter organization and different promoter selectivity.

## Discussion

We decided to focus on the alphaproteobacteria for a couple of reasons. First, there are previous reports that have shown that in this class some genomes have more than one *rpoH* gene (Narberhaus et al., 1997; Green and Donohue, 2006; Martinez-Salazar et al., 2009a). Secondly, previous phylogenomic analyses have established the species tree of some species from this class (Gupta and Mok, 2007; Castillo-Ramirez and Gonzalez, 2008) and this was of paramount importance for some part of the analyses that we carried out. The data set that we gathered covers the main orders of the alphaproteobacteria. In order to better understand the origins of the *rpoH* homologs, we used fully sequenced genomes, which have the virtue of providing an entire and unbiased view of the number of *rpoH* copies within each strain. To the best of our knowledge, this is the first study that has tried to establish the evolutionary forces that have molded the evolutionary history of any sigma factor.

It is a commonplace occurrence in alphaproteobacteria to have more than one *rpoH* homologs per genome. We found that 53% of the alphaproteobacteria genomes have two (or more) *rpoH* homologs and only few of them presented just one homolog. This is in agreement with previous experimental studies that have found that bacteria, such as *R. etli* (Martinez-Salazar et al., 2009a) or *R. sphaeroides* (Green and Donohue, 2006), have two members of the RpoH family. There are two possible explanations for the occurrence of extra *rpoH* homologs in a genome: they could have been introduced by HGT or they could have been originated through duplication. Our phylogenetic analysis shows that both processes have shaped the evolutionary history of *rpoH*. We note that most the *rpoH* homologs fall within two major groups and, given that the species relationships within each group is similar to that of the species tree—apart from the HGT cases-, we think that an ancient gene duplication is the most parsimonious explanation. Even more, our topology test analysis further reinforces this possibility. It is interesting to note that, according to our estimates, the ancient duplication occurred some time (95% HPD 1812–2491) after the Great Oxidation Event (the first significant increase in atmospheric oxygen) that is thought to have occurred around 2450–2320 mya (Bekker et al., 2004; Bekker and Kaufman, 2007)—even more so, considering that some *rpoH*_2_ genes are involved in the oxidative stress response (Martinez-Salazar et al., 2009a; Nuss et al., 2009; Dufour et al., 2012; Jans et al., 2013). However, the pattern generated by this ancient duplication has been peppered with HGT events. We did not detect any HGT between the gamma and alphaproteobacteria, which is expected given that this gene is highly connected and recently it has been shown that high gene connectivity curbs HGT (Cohen et al., 2011). However, we did find some events of HGT within the alphaproteobacteria and more specifically within the *rpoH*_1_ group.

While *rpoH*_1_ is clearly involved in heat-shock stress, experimental studies have shown that *rpoH*_2_ is related to other stress conditions in different bacteria; for instance, in *R. sphaeroides rpoH*_2_ has been implicated in oxygen stress (Dufour et al., 2012), while in *R. etli* (Martinez-Salazar et al., 2009a) it has been shown to be involved in the osmotic stress. Although RpoH_2_ proteins have shown not to be essential, they are bound to be relevant in the environment in which these bacteria dwell; along these lines we found that most of them are located on the chromosome and not on plasmids. Furthermore, in the case of *R. etli* it is known that genes that code for RpoH_1_ and RpoH_2_ belong to the core genome (Rosa Isela Santamaría personal communication). Notably, as far as the gammaproteobacteria are concerned, only one *rpoH* homolog per genome was found, suggesting that duplication events or even HGT introducing new *rpoH* genes have not been successful. Another salient trend that emerges from our analysis is the fact that *rpoH* homologs have been differentially conserved in each group. Clearly, whereas almost all the alphaproteobacteria genomes here analyzed have *rpoH*_1_ homologs, not all of them present *rpoH*_2_ homologs; in this regard, both the *Sphingomonadales* and the *Rickettsiales* only have *rpoH*_1_. Therefore, these gene loss events only affected the *rpoH*_2_ homologs and we think this also reflects the more essential nature of *rpoH*_1_ homologs. Of note, there was only one genome, *Rickettsia bellis* OSU, for which it was not possible to find either of the *rpoH* genes. Although the BLAST search found a hit for *rpoH*_1_ group, this hit did not pass our alignment criteria and therefore was not considered in our analysis. The presence of just *rpoH*_1_ in the *Rickettsiales* is not unexpected, as this group of obligate intracellular bacteria is known to have endured severe reductive genome processes (Darby et al., 2007; Renvoise et al., 2011).

Our selection analysis indicates that for most of the history of *rpoH* selection has acted to eliminate non-synonymous mutations—most of which are likely to be deleterious. Remarkably, we did not find evidence for sites under positive selection nor did we find evidence for a change in the amino acid evolutionary rate over time. Hence, the strong level of purifying selection is conserved over time. This notion that purifying selection is the main form of selection acting on this gene agrees with the findings that *rpoH*_1_ and *rpoH*_2_ are able to complement the temperature sensitivity of an *E. coli rpoH* mutant (Narberhaus et al., 1997; Green and Donohue, 2006; Martinez-Salazar et al., 2009a); in other words, selection has purged many of the changes that affect the protein initial function and, therefore, even *rpoH*_2_, which has been implicated in several stress conditions, is able to perform the function of the *E. coli rpoH*. However, we note that the level of purifying selection is variable and that some parts of the gene are more conserved than others. For instance, the region that codifies for the RpoH box (red dots, Figure 3)—a characteristic sequence of amino acid that is found in σ^32^ homologs—is under strong purifying selection and so does the region 2.4 (blue dots, Figure 3), which interacts with −10 motif of RpoH-dependent genes. On the other hand, we found clear evidence of relaxed purifying selection in the region 4.2—this region interacts with the −35 motif of the RpoH-dependent genes. This might help to explain the fact that *rpoH*_1_ and *rpoH*_2_ control—aside from a set of overlapping genes—distinct sets of genes, if one assumes that these sets of genes have different promoters. Actually, the fact that region 4.2 has accumulated more non-synonymous changes matches a recent finding by Barnett et al. (2012) in that study, when they looked at RpoH-dependent genes they found that the RpoH_1_-specific promoter sequence for the −35 and −10 motifs was CTTGAA-N_15−16_-CCTATAT, whereas that for RpoH_2_ was CTTGCC-N_15−16_-CCTATCT. If one compares the specific promoter sequences from that study, it is clear that for the −35 motif there are two differences (the last two adenines are changed to cytosines), while for the −10 motif there is only one difference (the last adenine is changed for a thymine). In other words, we found a more variable region 4.2 and comparing the −35 and −10 motifs of the RpoH-dependent genes, from Barnett et al. (2012), a more variable −35 motif is evident. Therefore, it might be that within the interaction between the region 4.2 and the −35 motif from the RpoH-dependent genes is where the differentiation to control different sets of genes lies. All in all, it seems that our selection analysis along with previous molecular studies—showing that different *rpoH* copies complement the *E. coli rpoH* mutant, but at the same time, these different copies control different sets of genes—suggest that there have been subtle protein functional changes between RpoHs encoded by different *rpoH* genes within a genome.

To understand how a gene evolves, it is of great significance to know how its coding sequence has changed over time, yet another important aspect is how the gene expression of this gene has changed over time. In our study as proxy for the latter aspect, we carried out an analysis to detect promoter motifs for both *rpoH*_1_ and *rpoH*_2_. Our promoter analysis suggests that *rpoH*_1_ and *rpoH*_2_ show different promoter selectivity. For instance, we note that whereas *rpoH*_1_ has promoter signals implying it is under transcriptional control of σ^70^ and σ^32^, *rpoH*_2_ only has promoter signals for σ^24^. We do not think that the results of our promoter analysis are spurious, as some previous experimental studies have characterized the promoters of the *rpoH* genes for some *Rhizobiales* species (Martinez-Salazar et al., 2009a; Schluter et al., 2013)—actually, that is why we chose this order to conduct our promoter analysis—and their findings agree with our promoter analysis. For instance, using 5′Rapid Amplification of cDNA Ends (RACE) to determine the transcription start sites of the *rpoH* genes in *R. etli* CFN42, it was found that *rpoH*_1_ is under transcriptional control of σ^70^, whereas *rpoH*_2_ shows promoter signals for σ^24^ (Martinez-Salazar et al., 2009a). Furthermore, another study also using 5′RACE determined that *rpoH*_2_ shows promoter signals for σ^24^ in *S. meliloti* 1021 (Schluter et al., 2013). Even experimental studies on other order (*Rhodobacterales*) support our findings, for example it has been shown that in *R. sphaeroides rpoH*_2_ is under the control of σ^24^ (Anthony et al., 2005; Dufour et al., 2008; Nuss et al., 2009) and thus further support the finding that *rpoH*_2_ has promoter signals for σ^24^. Interestingly, not only *rpoH*_1_ and *rpoH*_2_ have different potential promoters types but also these seem to be located at different distances in each gene. Our promoter analysis, supported by the experimental studies mentioned above, indicates that *rpoH1* and *rpoH2* are differentially expressed. The general picture that emerges from our analyses is that after the duplication (peppered with cases of HGT and gene loss), rather than changing substantially the protein function, selection has modified the promoter regions so that the activity of these σ^32^ factors is differentially controlled at the transcriptional level. Notably similar trends have been described in fungi (Wapinski et al., 2007), where it seems that duplicated genes diverge more frequently in their regulation and much less frequently in the biochemical nature of their functions.

Our analyses have dealt with long-term evolution rather than short-term evolution; however, it is important to consider how these extra copies were generated in the first place. It is important to mention that gene duplication—oftentimes also called gene amplification—could have an adaptive role in dosage response to stressful conditions over short-term evolutionary scales (Kondrashov, 2012). We note that, besides the dosage response scenario, there are other possibilities that could account for the benefits of duplications (Fares, 2015); such is the case of mutational and regulatory robustness (Fares et al., 2013; Keane et al., 2014; Fares, 2015). Remarkably, gene duplication has been often observed playing a part in heavy-metal tolerance, drug-resistance, and survival in stressful environments not only in bacteria but also in eukaryotes (Kondrashov, 2012). For instance, when six lines of *E. coli* were exposed to stressful high temperatures, several duplication and deletion events were identified (Riehle et al., 2001); notably, genes within the duplicated regions were related to stress and starvation conditions and the timing of these events was concurrent with an increase in the relative fitness of the strains. Hence, in order to better understand the origin of these extra copies and, therefore, have a more comprehensive view of the evolution of these sigma factors, we plan to conduct further research at much shorter evolutionary scales, such as those concerning recently emerged bacterial clones (Castillo-Ramirez et al., 2012) and recently originated species (Joseph et al., 2015).

In summary, the molecular evolution of the *rpoH* gene within the alphaproteobacteria appears to be shaped by an ancient duplication, with subsequent HGT and gene loss events, variable purifying selection across the gene and functional differentiation of the promoter regions. In a more general sense, by means of a phylogenomic approach, we were able to decipher some of the major evolutionary drivers of an important alternative sigma factor. This work shows that with the huge amount of genomes publicly available studies focusing on evolution of sigma factors are feasible and desirable to fully understand not only the nature of them but also, and more importantly, the complex net of bacterial gene regulation. We anticipate our study to be a point of reference for subsequent evolutionary studies of this and other sigma factor families.

## Author contributions

SC designed, supervised, and coordinated the study. GL conducted the blast searches, carried out the statistical model selection, and performed the promoter analysis. SC constructed the ML phylogenies, carried out the topology tests, conducted the molecular dating analysis, and ran the selection analysis as well as the Tajima's relative test. MC participated in the general discussion. SC and GL wrote the manuscript. MC critically revised the manuscript. All the authors reviewed and approved the manuscript.

## Funding

This work was supported by “Programa de Apoyo a Proyectos de Investigación e Innovación Tecnológica PAPIIT,” grant number IA200515 to SC. Funding for SC was also provided by the Programa de Genomica Evolutiva, CCG-UNAM.

### Conflict of interest statement

The authors declare that the research was conducted in the absence of any commercial or financial relationships that could be construed as a potential conflict of interest.
