# Supplementary material for: Evolution of a Sigma Factor: An All-In-One of Gene Duplication, Horizontal Gene Transfer, Purifying Selection, and Promoter Differentiation
Source: Front Microbiol. 2016 Apr 25;7:581. doi: 10.3389/fmicb.2016.00581 (PMC4843759; doi:10.3389/fmicb.2016.00581)
Supplement: Supplementary file 1 [file DataSheet1.pdf]

SUPPLEMENTARY DATA

Additional material 1 – genomes used for this study
Additional material 2 – orthologues used for the species tree
Additional material 3 – proxy for the species tree
Additional material 4 – common set of taxa used for the topology tests
Additional material 5 – Bayesian calibrated phylogeny
Additional material 6 – Rhizobiales genomes used for the promoter analysis
Additional material 7 – promoter analysis, spaced dyads analysis
Additional material 8 – promoter analysis, matrix-scan analysis
Additional material 9 – Trees using the common set of taxa used for the topology tests

Genomes	RpoHs protein Gi	Order
Xylella fastidiosa 9a5c	9107932	Outgroup Gammaproteobacteria
Pseudomonas aeruginosa PAO1	9946228	
Vibrio cholerae N16961	9654552	
Haemophilus influenzae Rd KW20	1573235	
Yersinia pestis CO92	115349424	
Escherichia coli K12	169890810	
Salmonella enterica typhimurium DT104	545009926	
Bartonella bacilliformis KC583	120614761, 120614628	RHIZOBIALES
Bartonella quintana str. Toulouse	49240203, 49239434	
Bartonella henselae str. Houston	49238984, 49237986	
Sinorhizobium meliloti 1021	187904236, 15075750	
Rhizobium leguminosarum bv. viciae 3841	115258988, 115258155	
Rhizobium etli CFN42	86283707, 86283042	
Agrobacterium radiobacter K84	221725177, 221724289	
Brucella abortus biovar str. 9-941	62196766, 62196658	
Mesorhizobium loti MAFF303099	14024055, 14023963	
Mesorhizobium sp. BNC1	110286692, 110286469	
Rhodopseudomonas palustris HaA2	86570609, 86570351	
Bradyrhizobium japonicum USDA 110	27355619, 27348550, 27353506	
Nitrobacter winogradskyi Nb-255	74421485	
Nitrobacter hamburgensis X14	91801253	
Rhodopseudomonas palustris BisA53	115516790	
Rhodopseudomonas palustris CGA009	39647290	
Rhodopseudomonas palustris BisB18	90104142	
Rhodopseudomonas palustris BisB5	91681296	
Agrobacterium tumefaciens C58	15157628	
Rhodobacter sphaeroides 2.4.1	77388590, 77387386	RHODOBACTERALES
Paracoccus denitrificans PD1222	119374715	
Roseobacter denitrificans OCh 114	109454642, 109455225	
Jannaschia sp. CCS1	88862946, 88865440	
Silicibacter sp. TM1040	99036577, 99036684	
Sphingomonas sp. MM-1	469478232	SPHINGOMONADALES
Sphingopyxis alaskensis RB2256	98977305	
Erythrobacter litoralis HTCC2594	84786145	
Zymomonas mobilis subsp. mobilis ZM4	56543219	
Novosphingobium aromaticivorans DSM 12444	87136738	
Magnetospirillum magneticum AMB-1	82946747, 82947127	RHODOSPIRILLALES
Acidiphilium cryptum JF-5	146403382	
Rhodospirillum rubrum F11	346717507, 346717599	
Rhodospirillum centenum SW	209958976, 209958920, 209958486	
Gluconobacter oxydans H24	411027919	
Rickettsia felis URRWXCal2	67004418	RICKETTSIALES
Rickettsia bellis OSU	N/A	
Ehrlichia chaffeensis str. Arkansas	88599975	
Anaplasma marginale str. St. Maries	56388037	
Neorickettsia sennetsu str. Miyayama	88600791	
Parvularcula bermudensis HTCC2503	303299057, 303299155	PARVULARCULALES
Caulobacter crescentus CB15	13424754	CAULOBACTERALES
Caulobacter segnis ATCC 21756	295430197	
Caulobacter sp. K31	167347060	
Brevundimonas subvibrioides ATCC 15264	302193017	
Phenylobacterium zucineum HLK1	196480465*, 196480703*, 196477295	
Asticcacaulis excentricus CB 48	315416792	

Additional material 1 – genomes used for this study

Genomes used in this study. All the sequences were dowloaded from the NCBI ftp site. The GI number is provided for each of the RpoH homologues found. The color coding is the same as in figure 1. *Only these two genes are encoded in plasmid, the rest of RpoH homologues are in chromosome.

Gene product	Gi	Phi test *p*-value
Ribosomal protein L5	169890663	
Predicted GTPase	169891454	
Phenylalanine-tRNA synthethase alpha subunit	169889208	
Arginyl-tRNA synthetase	169889367	
30S ribosomal protein S12	499898484	
30S ribosomal subunit protein S7	169890694	
30S ribosomal subunit protein S2	169887640	
Ribosomal protein L11	169891278	
50S ribosomal subunit protein L1	169891279	
**RNA polymerase, beta subunit**	**169891282**	**1**
Ribosomal protein L3	169890674	
50S ribosomal subunit protein L22	169890669	
30S ribosomal protein S3	446452088	
50S ribosomal subunit protein L14	169890665	
**50S ribosomal subunit protein L5**	**169890663**	**0.86**
**30S ribosomal subunit protein S8**	**169890661**	**0.99**
**Ribosomal protein L6P/L9E**	**915886771**	**0.99**
30S ribosomal subunit protein S5	169890658	
30S ribosomal subunit protein S13	169890653	
**30S ribosomal subunit protein S11**	**169890652**	**0.97**
50S ribosomal subunit protein L13	169890598	
30S ribosomal subunit protein S9	169890597	
**Seryl-tRNA synthetase**	**169888386**	**1**
30S ribosomal protein S15	835807966	
30S ribosomal subunit protein S17	169890666	
Ribosomal protein L15	169890656	
**RNA polymerase, alpha subunit**	**169890650**	**1**
Ribosomal protein L18	169890659	
Leucyl-tRNA synthetase	169888056	
**Ribosomal protein S4**	**169890651**	**0.98**
Threonylcarbamoyladenine synthase TsaD	485789200	

Additional material 2 – orthologues used for the species tree
List of the 31 ortologues, and their Gi numbers, used by Cicarelli and colleagues (Ref 21 in the main text) to construct the tree of life. We excluded 23 of the 31 orthologues because they either had more than one copy per genome (green) or were absent at least in one genome of our data set (red). We used the rest 8 orthologues (white rows and bold letters) for our proxy of the species tree. Of note, none of them had signals for recombination (see the p-values for the Phi test).

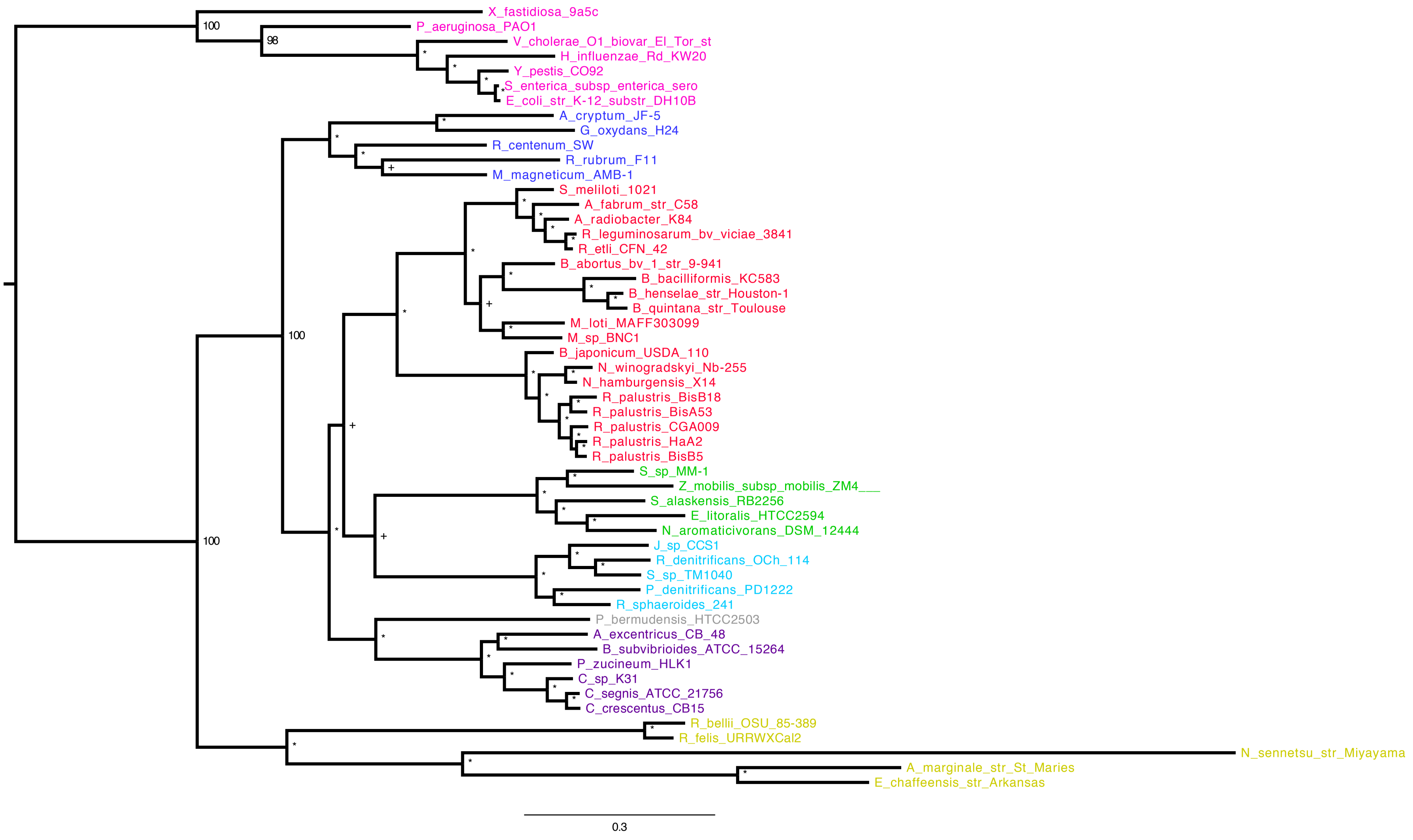

Additional material 3 – proxy for the species tree

Bartonella bacilliformis KC583
Bartonella quintana str. Toulouse
Bartonella henselae str. Houston
Sinorhizobium meliloti 1021
Rhizobium leguminosarum bv. viciae 3841
Rhizobium etli CFN42
Agrobacterium radiobacter K84
Brucella abortus biovar str. 9-941
Mesorhizobium loti MAFF303099
Mesorhizobium sp. BNC1
Rhodobacter sphaeroides 2.4.1
Roseobacter denitrificans OCh 114
Jannaschia sp. CCS1
Siliabacter sp. TM1040
Parvularcula bermudensis HTCC2503

Additional material 4 – common set of taxa used for the topology test
Common set of species used for the topology test. Each one of these species has two copies of *rpoH,* one in $rpoH_1$ and one in $rpoH_2$.

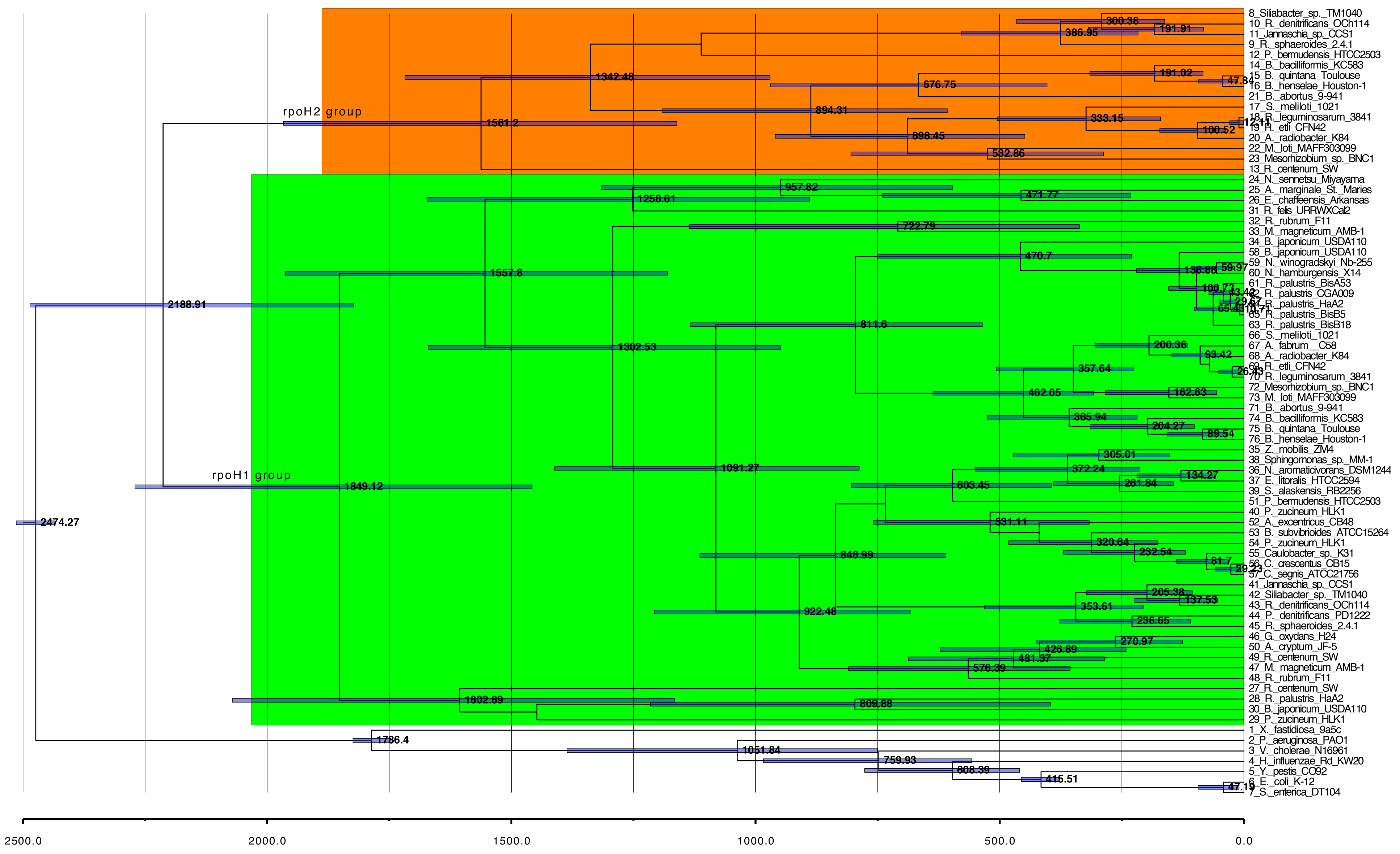

Additional material 5 – Bayesian calibrated phylogeny

Genome	RpoH1 Gi number	RpoH2 Gi number
R. etli CFN42	86283042	86283707
S. fredii USDA257	398355004	398355529
S. fredii HH103	378827322	378827869
S. fredii NGR234	227823295	227823777
S. meliloti SM11	384537310	384537795
S. meliloti Rm41	407721792	407722266
S. meliloti GR4	433614558	433615032
S. meliloti BL225C	384530609	384531080
S. meliloti AK83	334317483	334317955
S. meliloti 2011	470189691	470190229
S. meliloti 1021	15966479	195970121
S. medicae WSM419	150397812	150398305
A. vitis S4	222149598	222150148
R. etli CIAT 652	190893168	190893879
R. leguminosarum bv. trifolii WSM2304	209550663	209551384
R. etli bv. mimosae str. Mim1	528830121	528830807
R. leguminosarum bv. trifolii WSM1325	241206034	241206826
R. leguminosarum bv. viciae 3841	116253507	116254340
A. radiobacter K84	222086841	222087729
R. tropici CIAT 899	440227629	440228410

Additional material 6 – Rhizobiales genomes used for the promoter analysis
List of genomes used for the promoter analysis. The Gi number is shown for each sequence of each *rpoH* group.

Motif in *$rpoH_1$* upstream regions

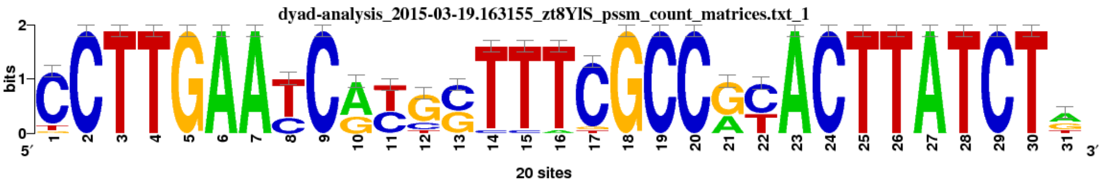

Motif in *$rpoH_2$* upstream regions

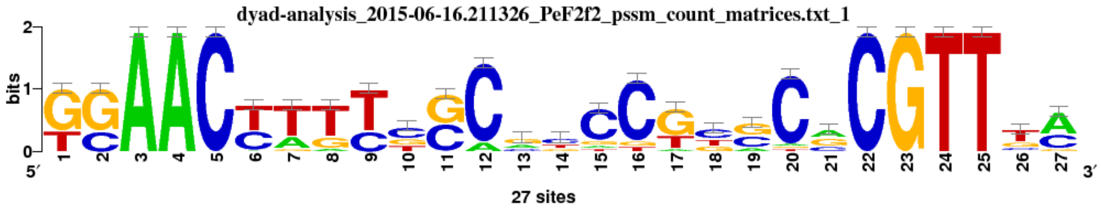

Additional material 7 – promoter analysis, spaced dyads analysis

Motifs identified by *de novo* promoter prediction. The logos were generated by RSAT suit as a result of the analysis of spaced dyads (see methods). The height of each nucleotide is given in bits and represents the relative contribution of that nucleotide to that position.

Genome	Gene	-35 Box	Spacer	-10 Box	Distance from ATG (nt)	Strand	Replicon	Promoter class
Rhizobium etli CFN 42	RHE_CH03342	CTTGAA	CCGCGGTTTCGCCATACTTATCT	GTATAT	105	+	NC_007761	SigA
Sinorhizobium meliloti SM11	SM11_chr2889	CTTGAA	TCATGCTTTCGCCGCACTTAT	CTATTA	80	+	NC_017325	SigA
Sinorhizobium meliloti Rm41	BN406_02583	CTTGAA	TCATGCTTTCGCCGCACTTAT	CTATTA	80	+	NC_018700	SigA
Sinorhizobium meliloti GR4	C770_GR4Chr2850	CTTGAA	TCATGCTTTCGCCGCACTTAT	CTATTA	80	+	NC_019845	SigA
Sinorhizobium meliloti BL225C	SinmeB_2557	CTTGAA	TCATGCTTTCGCCGCACTTAT	CTATTA	80	+	NC_017322	SigA
Sinorhizobium meliloti AK83	Sinme_2781	CTTGAA	TCATGCTTTCGCCGCACTTAT	CTATTA	80	+	NC_015590	SigA
Sinorhizobium meliloti 2011	SM2011_c00646	CTTGAA	TCATGCTTTCGCCGCACTTAT	CTATTA	80	+	NC_020528	SigA
Sinorhizobium meliloti 1021	SMc00646	CTTGAA	TCATGCTTTCGCCGCACTTAT	CTATTA	80	+	NC_003047	SigA
Sinorhizobium medicae WSM419	Smed_2614	CTTGAA	TCATGCTTTCGCCGCACTTAT	CTATTA	80	+	NC_009636	SigA
Agrobacterium vitis S4	Avi_3538	CTTGCC	CGTTTGGCATTGGGAAGC	TCATGG	17	+	NC_011989	SigA
Rhizobium etli CIAT 652	RHECIAT_CH0003586	CTTGAA	CCGCCGTTTCGCCATACTTATCT	GTATAT	106	+	NC_010994	SigA
Rhizobium leguminosarum bv. trifolii WSM2304	Rleg2_3087	CTTGAA	CCGCTGTTTCGCCACACTTATCT	GTATAT	106	+	NC_011369	SigA
Rhizobium etli bv. mimosae str. Mim1	REMIM1_CH03402	CTTGAA	CCGCGGTTTCGCCATACTTATCT	GTATGT	104	+	NC_012850	SigA
Rhizobium leguminosarum bv. trifolii WSM1325	Rleg_3344	CTTGAA	CCGCCGTTTCGCCACA	GTATGT	105	+	NC_012850	SigA
Rhizobium leguminosarum bv. viciae 3841	RL3766	CTTGAA	CCGCCGTTTCGCCACACTTATCT	GTATAT	106	+	NC_008380	SigA
Agrobacterium radiobacter K84	Arad_3513	CTTGAA	TCACTGTTTGGCCGTACTTATCT	ACATAT	107	+	NC_011985	SigA
Rhizobium etli CFN 42	RHE_CH03342	CTTGAA	CCGCGGTTTCGCCATA	CTTATCT	111	+	NC_007761	RpoH
Sinorhizobium fredii USDA 257	USDA257_c51860	CTTGAA	TCATGCTTTCGCCGTA	CTTATCT	80	+	NC_018000	RpoH
Sinorhizobium fredii HH103	SFHH103_02735	CTTGAA	TCATGCTTTCGCCGTA	CTTATCT	81	+	NC_016812	RpoH
Sinorhizobium fredii NGR234	NGR_c27660	CTTGAA	TCATGCTTTCGCCGTA	CTTATCT	81	+	NC_012587	RpoH
Sinorhizobium meliloti SM11	SM11_chr2889	CTTGAA	TCATGCTTTCGCCGCA	CTTATCT	84	+	NC_017325	RpoH
Sinorhizobium meliloti Rm41	BN406_02583	CTTGAA	TCATGCTTTCGCCGCA	CTTATCT	84	+	NC_018700	RpoH
Sinorhizobium meliloti GR4	C770_GR4Chr2850	CTTGAA	TCATGCTTTCGCCGCA	CTTATCT	84	+	NC_019845	RpoH
Sinorhizobium meliloti BL225C	SinmeB_2557	CTTGAA	TCATGCTTTCGCCGCA	CTTATCT	84	+	NC_017322	RpoH
Sinorhizobium meliloti AK83	Sinme_2781	CTTGAA	TCATGCTTTCGCCGCA	CTTATCT	84	+	NC_015590	RpoH
Sinorhizobium meliloti 2011	SM2011_c00646	CTTGAA	TCATGCTTTCGCCGCA	CTTATCT	84	+	NC_020528	RpoH
Sinorhizobium meliloti 1021	SMc00646	CTTGAA	TCATGCTTTCGCCGCA	CTTATCT	84	+	NC_003047	RpoH
Sinorhizobium medicae WSM419	Smed_2614	CTTGAA	TCATGCTTTCGCCGCA	CTTATCT	84	+	NC_009636	RpoH
Agrobacterium vitis S4	Avi_3538	CTTGAA	TCGCGGCCATGCCACA	CTTATCT	90	+	NC_011989	RpoH
Rhizobium etli CIAT 652	RHECIAT_CH0003586	CTTGAA	CCGCCGTTTCGCCATA	CTTATCT	113	+	NC_010994	RpoH
Rhizobium leguminosarum bv. trifolii WSM2304	Rleg2_3087	CTTGAA	CCGCTGTTTCGCCACA	CTTATCT	112	+	NC_011369	RpoH
Rhizobium etli bv. mimosae str. Mim1	REMIM1_CH03402	CTTGAA	CCGCGGTTTCGCCATA	CTTATCT	111	+	NC_021905	RpoH
Rhizobium leguminosarum bv. trifolii WSM1325	Rleg_3344	CTTGAA	CCGCCGTTTCGCCACA	CTTATCT	111	+	NC_012850	RpoH
Rhizobium leguminosarum bv. viciae 3841	RL3766	CTTGAA	CCGCCGTTTCGCCACA	CTTATCT	111	+	NC_008380	RpoH
Agrobacterium radiobacter K84	Arad_3513	CTTGAA	TCACTGTTTGGCCGTA	CTTATCT	113	+	NC_011985	RpoH
Rhizobium tropici CIAT 899	RTCIAT899_CH13980	CTTGAA	TCACCGTTTCGCCGTA	CTTATCT	115	+	NC_020059	RpoH
Rhizobium etli CFN 42	RHE_CH03342	GGAACA	AAAGCCGCACGTCC	CGGTTC	62	+	NC_007761	RpoE
Agrobacterium radiobacter K84	Arad_3513	GGACCA	AATCTCGAATAGGAGGGTGC	ACATTC	1	+	NC_011985	RpoE

Additional material 8 – promoter analysis, matrix-scan analysis

The matrices to identify signals promoters in the upstream regions of *rpoH*1 genes group were built using info-gibbs (see methods). The headings of each column show the details of each promoter.

Genome	Gene	-35 Box	Spacer	-10 Box	Distance from ATG (nt)	Strand	Replicon	Promoter class
Sinorhizobium fredii HH103	SFHH103_03284	GGAACT	TTTCGACCCCGCGC	ACGTTT	207	-	NC_016812	RpoE
Sinorhizobium fredii NGR234	NGR_c32660	GGAACT	TTTCGACCCTGCGC	ACGTTT	207	-	NC_012587	RpoE
Sinorhizobium meliloti SM11	SM11_chr3384	GGAACT	TTTCGCGCCCGCGC	ACGTTT	212	-	NC_017325	RpoE
Sinorhizobium meliloti Rm41	BN406_03057	GGAACT	TTTCGCGCCCGCGC	ACGTTT	212	-	NC_018700	RpoE
Sinorhizobium meliloti GR4	C770_GR4Chr3351	GGAACT	TTTCGCGCCCGCGC	ACGTTT	212	-	NC_019845	RpoE
Sinorhizobium meliloti BL225C	SinmeB_3028	GGAACT	TTTCGCGCCCGCGC	ACGTTT	212	-	NC_017322	RpoE
Sinorhizobium meliloti AK83	Sinme_3253	GGAACT	TTTCGCGCCCGCGC	ACGTTT	212	-	NC_015590	RpoE
Sinorhizobium meliloti 2011	SM2011_c03873	GGAACT	TTTCGCGCCCGCGC	ACGTTT	212	-	NC_020528	RpoE
Sinorhizobium meliloti 1021	SMc03873	GGAACT	TTTCGCGCCCGCGC	ACGTTT	212	-	NC_003047	RpoE
Sinorhizobium medicae WSM419	Smed_3111	GGAACT	TTTCCCGCCCGCGC	ACGTTT	210	-	NC_009636	RpoE
Agrobacterium vitis S4	Avi_4270	GGAACA	TTCCGCAGCTTTAA	GCGTTA	186	-	NC_011989	RpoE
Rhizobium leguminosarum bv. trifolii WSM2304	Rleg2_3813	GGAACT	TTTTCACGGCTGCC	GCGTTT	298	-	NC_011369	RpoE
Rhizobium leguminosarum bv. trifolii WSM1325	Rleg_4142	GGAACT	TTTTGCCGACTGCC	GCGTTT	297	-	NC_012850	RpoE
Rhizobium leguminosarum bv. viciae 3841	RL4614	GGAACT	TTTTCCCGACTGCC	GCGTTT	246	-	NC_008380	RpoE
Agrobacterium radiobacter K84	Arad_4669	GGAACC	TTTTGCGATCTGCC	GCGTTT	322	-	NC_011985	RpoE
Rhizobium tropici CIAT 899	RTCIAT899_CH17965	GGAACC	TTTTCCGTTCTGAT	GCGTTT	308	-	NC_020059	RpoE
Rhizobium tropici CIAT 899	RTCIAT899_CH17965	CTTGCT	GCCCTTTTCGTCGACCGCG	GCATTA	34	-	NC_020059	SigA

Additional material 8 – promoter analysis, matrix-scan analysis

The matrices to identify signals promoters in the uptream regions of *rpoH2* genes group were built by info-gibbs (see methods). The headings of each column show the details of each promoter.

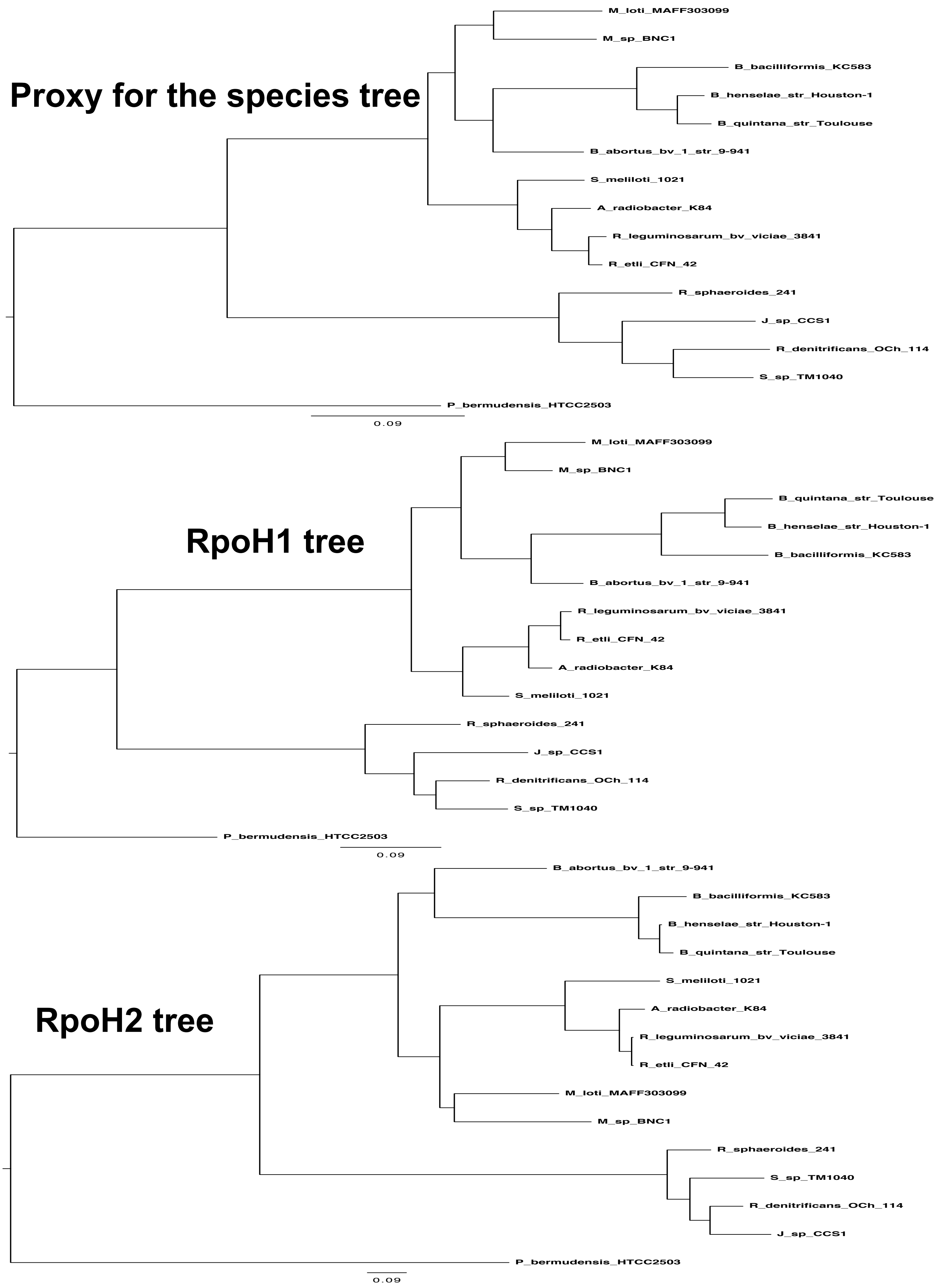

Additional material 9 – Trees with the common set of taxa
